# Low expression of novel lncRNA RP11-462C24.1 suggests a biomarker of poor prognosis in colorectal cancer

**DOI:** 10.1007/s12032-014-0031-7

**Published:** 2014-06-08

**Authors:** Debing Shi, Hongtu Zheng, Changhua Zhuo, Junjie Peng, Dawei Li, Ye Xu, Xinxiang Li, Guoxiang Cai, Sanjun Cai

**Affiliations:** 1 Department of Colorectal Surgery, Fudan University Shanghai Cancer Center, Shanghai, 200032 China; 2 Department of Oncology, Shanghai Medical College, Fudan University, Shanghai, 200032 China

**Keywords:** Colorectal cancer, Long noncoding RNAs, Microarray, RP11-462C24.1, Survival

## Abstract

Long noncoding RNAs (lncRNAs) have recently emerged as a major class of regulatory molecules, which were involved in a broad range of biological processes and complex diseases. Research on lncRNAs may shed light on tumorigenesis and progression of colorectal cancer (CRC). The purpose of the present study was to identify lncRNAs correlated with CRC and then investigate their potential functions. We selected 92 patients for this prospective study and then collected the tumor samples and clinical records. First, the global lncRNA expression profiles in tumor and adjacent normal tissues of patients with non-metastatic CRC and patients with metastatic CRC were measured by microarray assay. Then, a noteworthy lncRNAs RP11-462C24.1 whose function was previously unknown was explored in detail on the aspect of the association of its expression level and clinicopathological features of CRC and patients’ survival. We found that RP11-462C24.1 expression level was lower in cancer tissues compared with adjacent normal samples (*P* < 0.001). Furthermore, its expression level was lower in CRC patients with metastasis than those without metastasis (*P* = 0.049). That is, RP11-462C24.1 expression level decreased as the malignant degree of CRC increased. In addition, low expression of RP11-462C24.1 significantly correlated with more distant metastasis (*P* = 0.011). The areas under ROC curves were 0.78 and 0.65 for RP11-462C24.1, distinguishing CRC from normal tissue and distinguishing CRC without metastasis from CRC with metastasis, respectively. Multivariate analysis identified that RP11-462C24.1 was an independent predictor for patients prognosis (*P* = 0.005). Furthermore, Kaplan–Meier analysis showed that patients with low expression of RP11-462C24.1 had a poor disease-free survival (*P* < 0.001). This is the first study that correlates RP11-462C24.1 expression profile with malignancy grade in human CRC. Our results showed that RP11-462C24.1 could be a potential novel prognostic marker for CRC, and thus, provided a new strategy for CRC diagnosis. Meanwhile, our findings indicated the potential roles of RP11-462C24.1 in tumorigenesis and progression of CRC, which gave a clue for future studies.

**Electronic supplementary material:**

The online version of this article (doi:10.1007/s12032-014-0031-7) contains supplementary material, which is available to authorized users.

## Introduction

Colorectal cancer (CRC), which ranks the third in the cancer morbidity and the second in the cancer mortality, is the most prevalent malignant cancer in the world with annual new cases exceeding 1,000,000 [1]. Although great progresses have been made in therapy of CRC, 30–40 % patients still died of relapse and metastasis, among which liver metastasis is the leading cause of death [2–4]. Therefore, it is crucial to accurately diagnose liver metastasis in early stage and then following a preventive treatment. Currently, the diagnosis for relapse and metastasis and therapy strategies are mostly based on tumor node metastasis (TNM) stages [5, 6]. However, this method always leads to a misdiagnosis, as it excludes the underlying molecular mechanisms, which were responsible for cancer progression [7]. Thus far, more and more studies are looking for biomarkers to employ them in the early diagnosis of liver metastasis and accurate prognosis for patients with CRC.

Long noncoding RNA (lncRNA) is a type of RNA molecule with length of more than 200 bp and lacks an open reading frame of significant length and the capability of coding protein [8–11]. When it comes to the structural similarity to mRNA, both kinds of RNAs are marked by 5′ cap and 3′ poly A. However, it has a diverse subcellular location and plays important roles in many aspects of cell activity. As a new type of regulatory RNA molecule, lncRNAs exert the role of regulating gene expression in various layers of epigenetics, transcription, post-transcription and translation during the development of cancer [12, 13]. Based on their genomic proximity to protein-coding genes, lcnRNAs are mainly classified into five types: sense, antisense, bidirectional, intronic, and intergenic [10, 14].

Cancer is a complex disease involving multiple changes in gene expression. To date, researchers are mainly interested in protein-coding genes, which indeed have been shown to affect cancer progression. However, the underlying mechanisms of cancer are far from complete elucidation. With the discovery of lncRNA, it provided a new insight into revealing uncharacterized aspects of cancer biology. Recently, several studies have demonstrated that lncRNAs are not only involved in the origin and development of human diverse cancers, but also as potential biomarkers for the prognosis for CRC patients. HOX Antisense Intergenic RNA (HOTAIR) was reported to be a negative prognostic factor for breast, colon, liver, and pancreatic cancer patient survival and involved in chromatin remodeling by interaction with polycomb repressive complex 2 (PRC2) [15–19]. Metastasis-associated lung adenocarcinoma transcript 1(MALAT1), also known as NEAT2 (nuclear-enriched abundantly transcript 2), was found to be overexpressed not only in early-stage metastasizing non-small cell lung cancer (NSCLC) but also in breast, pancreas, colon, prostate, and liver cancers [20, 21]. It was involved in regulated alternative splicing and transcriptional activity [22, 23]. More important, patients with high expression level of MALAT1 have poor prognosis [21]. Colorectal neoplasia differentially expressed (CRNDE), an up-regulated lncRNA in colorectal cancer, glioma, and leukemia, was reported with complex alternative splicing and multifunctional in tumorigenesis [24–27]. However, the relationship between lncRNA expression level and progression of CRC is still elusive.

In the present study, we aim to find new lncRNAs that correlated with CRC and test whether the expression of those lncRNA could be a potential biomarker for CRC. Specifically, we first want to identify all aberrantly expressed lncRNAs in CRC, by applying microarray assay. Second, we further investigated the expression pattern and genomic location of these dysregulated lncRNAs. Then, six aberrantly lncRNAs (ENST00000428029, ENST00000423943, RP11-462C24.1, AK097793, ENST00000393516 and uc002wvk.2) were selected and further confirmed their expression pattern by RT-PCR assay. Finally, we focused on one lncRNA, RP11-462C24.1, which could be a biomarker for CRC, for the analysis of association between its expression and clinical characteristics and association between its expression and patients’ survival.

## Materials and methods

### CRC samples and clinical data collection

A total of 92 patients were selected in this prospective study. Six of them were involved in a microarray assay to figure out lncRNAs aberrantly expressed in CRC. Specimens underwent resection of the primary CRC at Fudan University Shanghai Cancer Center. All the diagnoses of CRC were histopathologically confirmed. No patient has received preoperative treatment. Resected tissue samples were immediately frozen in liquid nitrogen and stored at −80 °C until RNA extraction. The data collected on all subjects, including age, gender, disease-specific survival (DSS) and CRC features, such as tumor size, location, histological stage, depth of invasion, and the status of liver metastasis; detailed information were given in Supplementary Material 1. Clinical stage of CRC was evaluated based on the TNM classification system [28, 29]. Patients follow-up were performed every 2–3 months during the first year after surgery and 3–6 months thereafter until November 30, 2012. All the 92 patients had completed follow-up, and 21 patients died of CRC. The DSS was defined as the length of time between the surgery and death specifically from the cancer.

### Ethics statement

This study was conducted according to the principles expressed in the Declaration of Helsinki. Tissue specimen collections were made with full informed consent of the patients and following institutional ethical guidelines that were reviewed and approved by the ethics committee at the hospital clinical ethics committee (ID 050432-4-1212B), Fudan University, Shanghai, China.

### Microarray assay

Six patients were involved in microarray assay. Of which, three patients were diagnosed as CRC without metastasis, whereas other three patients were diagnosed as CRC with liver metastasis. Specifically, cancer tissues from three patients with non-metastatic CRC were pooled and hybridized to one chip. Matched adjacent normal tissues from the above identical three patients were pooled and hybridized to second chip. Cancer tissues from the remaining three patients with non-metastatic CRC were pooled and hybridized to third chip. It thus allowed us to roughly detect the dysregulated lncRNAs between CRC tumor tissues and adjacent normal tissues and between CRC without metastasis and CRC with metastasis. Details about pooling information are described in Supplementary Material 2. Total RNA was extracted using TRIzol (Invitrogen, Carlsbad, CA, USA), following the manufacturers’ protocol. RNA preparation was cleaned up by RNeasy^®^ MinElute™ Cleanup Kit (Qiagen, Valencia, CA, USA). RNA integrity was assessed by standard denaturing agarose gel electrophoresis. One micro liter of total RNA from each sample was amplified and transcribed into fluorescent cRNA along the entire length of the transcripts without bias utilizing a random priming method, and cRNA was labeled and hybridized to the Human lncRNA Array v2.0 (8 × 60 K, Arraystar). In total, 33,045 lncRNAs and 30,215 coding transcripts, which were collected from the most authoritative databases such as Reference Sequence, University of California, Santa Cruz (UCSC), Knowngenes, Ensembl and many related literatures can be detected by the microarray. After washing slides, the arrays were scanned by the Agilent Scanner G2505B and the acquired array images were analyzed by Agilent Feature Extraction software (version 10.7.3.1). Quantile normalization and subsequent data processing were performed using the GeneSpring GX v11.5.1 software package (Agilent Technologies).

### Quantitative real-time PCR

To further confirm the generality of expression pattern of six lncRNAs in human population, specimens from 86 patients, who comprised of 36 patients without metastatic CRC and 50 patients with metastatic CRC, were used to perform qRT-PCR assay. Total RNA was extracted from frozen specimens using TRIzol reagent (Invitrogen Life Technologies). The 20 μL RT reactions were performed using a PrimeScript^®^ RT reagent Kit (Takara, Dalian, China) and incubated for 30 min at 37 °C, 5 s at 85 °C and then maintained at 4 °C. For real-time PCR, 1 μL diluted RT products were mixed with 10 μL of 2× SYBR^®^ Premix Ex Taq™ (Takara, Dalian, China), 0.6 μL forward and reverse primers (10 μM), and 8.4 μL nuclease-free water in a final volume of 20 μL, according to manufacturer’s instructions. The primers used in this study are included in Supplementary Material 3. All reactions were run on the Eppendorf Mastercycler EP Gradient S (Eppendorf, Germany), using the following protocol: one cycle at 95 °C for 3 min; 40 cycles of 95 °C for 15 s, and 60 °C for 60 s. The specificity of the PCR amplification was validated by the presence of a single peak in the melting curve analyses. Each RT-qPCR experiment was repeated three times. Relative expression of genes was calculated using the comparative cycle threshold (CT) (2^−ΔΔCT^) method with glyceraldehyde-3-phosphate dehydrogenase (GAPDH) as the endogenous control.

### Statistical analysis

All statistical analyses were performed using R software (http://www.r-project.org/). Correlation between RP11-462C24.1 expression and clinical factors was tested using χ^2^ tests. Univariate analysis was used to explore clinicopathological factors (i.e., age, gender, tumor size, tumor stage, invasion length) related with disease-specific survival. Then, significant clinicopathological factors, which were measured in univariate analysis, were further selected for multivariate analysis to reduce related factors. Cox proportional hazards analysis was applied to calculate the hazard ratio (HR) and the 95 % confidence interval (CI). In addition, the Kaplan–Meier survival analysis was applied to test the correlations between expression level and patients’ survival. The log-rank test was performed to test the statistical differences between survival curve of patients with lower RP11-462C24.1 expression level and that of patients with higher RP11-462C24.1 expression level. The mean value was used as classification threshold when necessary in χ^2^ tests and univariate/multivariate analysis. A two-tailed *P* value of 0.05 or less was considered statistically significant.

## Results

### Overview of lncRNAs expression profile in CRC

To identify lncRNAs specifically dysregulated in CRC, we firstly compared expression profile between CRC tissues and adjacent normal tissues. A total of 5,963 lncRNAs demonstrated differential expressions (fold change ≥2) between tumor tissues and adjacent normal tissues from non-metastatic CRC patients. In total, 3,029 lncRNAs were over-expressed, whereas 2,934 lncRNAs were down-regulated in CRC tumor tissues. The AC092165.4 was the most over-expressed lncRNA and followed by AP000525.8, PNAS-108, lincRNA-NANOS3-2, BC016035, AK057037 and AL137280. However, the AK024585 was the most down-regulated lncRNAs and followed by RP11-561O23.7, BC032913, AC007225.1, RP11-183K14.2, AC009133.1, and DQ597482. The detailed information is in Table 1.

**Table 1:** Parts of differentially expressed lncRNAs between CRC tissues and adjacent normal tissues and between metastatic and non-metastatic CRC samples

GeneSymbol	Regulation	Fold change
Non-metastatic CRC versus adjacent sample		
AC092165.4	Up	131
AP000525.8	Up	100
PNAS-108	Up	50
lincRNA-NANOS3-2	Up	30
BC016035	Up	29
AK057037	Up	28
AL137280	Up	24
AL137281	Up	22
AK024585	Down	46
RP11-561O23.7	Down	43
BC032913	Down	32
AC007225.1	Down	30
RP11-183K14.2	Down	26
AC009133.1	Down	25
MTB	Down	25
DQ597482	Down	21
Metastatic versus non-metastatic CRC sample		
XIST	Up	445
TRIM78P	Up	75
RP11-753E22.3	Up	71
DQ597482	Up	68
XIST	Up	58
CH17-12M21.1	Up	50
RP11-598F17.1	Up	49
CFLP4	Up	48
RP11-397E7.1	Down	92
RP1-125N5.2	Down	35
AK098474	Down	32
BX322613.3	Down	31
AX747988	Down	29
RP11-543D5.3	Down	28
lincRNA-KLF4-4	Down	28
AC008625.2	Down	27

We further compared expression profile between CRC tissues with metastasis and CRC tissues without metastasis. In total, there were 30,605 lncRNAs, which shown different expression pattern, consisting of 27,549 up-regulated and 3,056 down-regulated lncRNAs. The XIST was the most up-regulated lncRNA and followed by TRIM78P, RP11-753E22.3, DQ597482, CH17-12M21.1, RP11-598F17.1, CFLP4, CFLP3, AL358913.1, VDAC1P4, CTD-2244C20.1, KRT8P25, and KRT8P7, whereas the RP11-397E7.1 was the most down-regulated lncRNA and followed by RP1-125N5.2, AK098474, BX322613.3, AX747988, RP11-543D5.3, lincRNA-KLF4-4, AC008625.2, lincRNA-BBOX1-2, AL137280, AK091806, BC016035, RP11-506D12.1, lincRNA-NANOS3-2, RP11-128M1.1, CTGLF11P, CR620692, and RP11-630I5.1. The detailed information for top 16 lncRNA, which showed significantly difference in expression, was provided in Table 1. The outputs from microarray trails have been submitted to the GEO database with accession No. GSE52413.

### RP11-462C24.1 was down-regulated in CRC

**Fig. 1:**
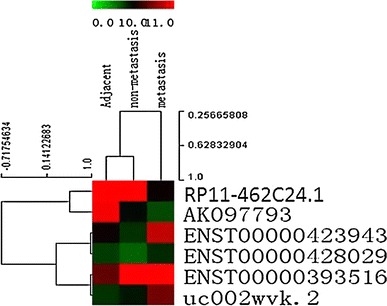
Unsupervised hierarchical clustering of samples with 6 lncRNAs

**Fig. 2:**
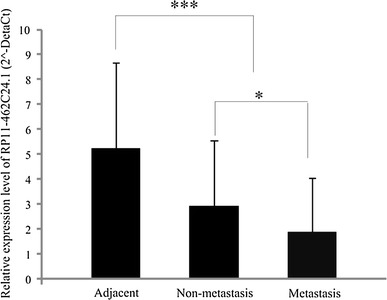
Relative expression of RP11-462C24.1 in metastatic, non-metastatic, and adjacent samples. RP11-462C24.1 expression was validated in 86 pairs of colorectal cancer and adjacent normal tissues by Q-RT-PCR, which was composed of 36 pairs of non-metastasis colorectal cancer samples and 50 pairs of metastasis samples. The height of the columns in the chart represent the mean expression value of 2^−Δ*C*t^ (Δ*C*_t_ = *C*_t_(RP11-46C24.1) − *C*_t_(GAPDH)); The *bars* represent standard errors. *P < 0.05, ***P < 0.001

Of all the differentially expressed lncRNAs indicated by lncRNA expression profiling, six lncRNAs (ENST00000428029, ENST00000423943, RP11-462C24.1, AK097793, ENST00000393516, and uc002wvk.2) were selected because of their expression levels and genomic locations. Unsupervised hierarchical clustering was constructed using MeV (Multi Experiment Viewer) v4.9 [30] to analyze the expression profile of three groups of samples (Fig. 1). Their expression levels were further confirmed in additional cohort of 86 samples, which comprised of 36 patients without metastatic CRC and 50 patients with metastatic CRC, using real-time PCR assay. Except ENST00000423943, other five lncRNAs showed the consistent pattern. We then focused on RP11-462C24.1, because it was down-regulated in CRC tissues compared with CRC adjacent normal tissues (*P* < 0.001) and even lower expressed in CRC tissues with metastasis than CRC tissues without metastasis (*P* = 0.049; Fig. 2), implying RP11-462C24.1 may play a role in tumor suppression.

### RP11-462C24.1 expression showed significant difference between CRC tumor tissues and adjacent normal tissue

**Fig. 3:**
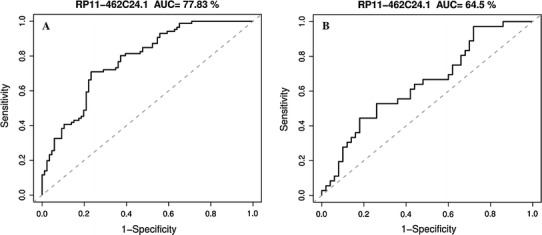
**a** ROC curve of patients with colorectal cancer based on RP11-462C24.1 expression in tumor tissues and adjacent normal tissues. **b** ROC curve of patients with colorectal cancer based on RP11-462C24.1 expression in metastatic tumor tissues and non-metastatic tumor tissues

To explore whether RP11-462C24.1 can serve as a biomarker to distinguish CRC from normal tissue, we constructed a receiver operating characteristic (ROC) curve by grouping all tumor samples, including samples with metastasis and without metastasis into one class and then grouping all normal samples into another class. RP11-462C24.1 expression level obtained from RT-PCR data of a cohort of 86 patients mentioned above. The areas under ROC curve (AUC) of this ROC are 0.78 (Fig. 3a). Then, we constructed a ROC using all tumor samples alone by taking CRC samples with metastasis as one group and CRC samples without metastasis as another group. AUC of this ROC are 0.65 (Fig. 3b). Both ROC curves implied RP11-462C24.1 has a potential diagnostic value in CRC.

### Correlation between RP11-462C24.1 expression and clinical characteristics

We assessed the correlations between RP11-462C24.1 expression and clinical characteristics by labeling the expression levels of RP11-462C24.1 in CRC tissue as low or high in relation to the mean value. RP11-462C24.1 expression level obtained from RT-PCR data of a cohort of 86 patients mentioned above. Of the 86 patients with CRC, 23 patients were classified into high RP11-462C24.1 group, and the remaining 63 patients were classified into low RP11-462C24.1 group. RP11-462C24.1 expression level was significantly correlated with distant metastasis (*P* = 0.011; Table 2). However, RP11-462C24.1 expression level was not significantly associated with other clinical factors, such as age (*P* = 0.132), gender (*P* = 0.300), tumor size (*P* = 0.180), depth of invasion (*P* = 0.372) or tumor stage (*P* = 0.070; Table 2).

**Table 2:** Correlations between RP11-462C24.1 expression and clinical characteristics

Characteristics	Number of case	RP11-462C24.1 expression				*P* value
		High (*n* = 32)	%	Low (*n* = 54)	%	
Age (years)	86					0.132
<60	43	13	40.6	30	55.6	
≥60	43	19	59.4	24	44.4	
Gender						0.300
Male	63	25	78.1	38	70.4	
Female	23	7	21.9	16	29.6	
Tumor size (cm)						0.180
<4	31	14	43.8	17	31.5	
≥4	55	18	56.2	37	68.5	
Depth of invasion						0.372
T1,T2	16	7	21.9	9	16.7	
T3,T4	70	25	78.1	45	83.3	
Tumor stage						0.070
I and II	23	12	37.5	11	20.4	
III and IV	63	20	62.5	43	79.6	
Metastasis						0.011*
Absent	36	19	59.4	17	31.5	
Present	50	13	40.6	37	68.5	

* *P* < 0.05

### Association between RP11-462C24.1 expression and patients’ survival

**Fig. 4:**
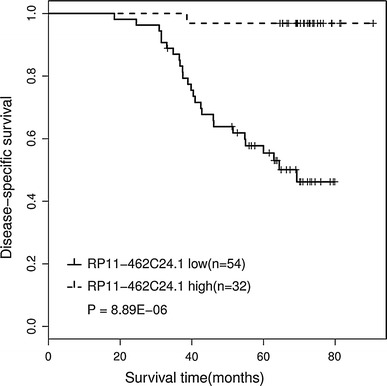
Kaplan–Meier survival curves of patients with colorectal cancer based on RP11-462C24.1 expression status

To explored factors responsible for patients’ survival, univariate and multivariate analysis were carried out. RP11-462C24.1 expression level obtained from RT-PCR data of a cohort of 86 patients mentioned above. Univariate analysis of disease-specific survival (DSS) revealed that metastasis (*P* = 0.0005), tumor stage (*P* = 0.015), and RP11-462C24.1 expression (*P* = 0.002) were prognostic indicators (Table 3). Multivariate analysis showed that the expression of RP11-462C24.1 (*P* = 0.005) and metastasis (*P* = 0.020) were independent prognostic indicators for survival of patients with CRC (Table 4). Furthermore, Kaplan–Meier analysis demonstrated that patients with low expression of RP11-462C24.1 had a significantly poor prognosis than those with high RP11-462C24.1 expression (*P* < 0.001; Fig. 4).

**Table 3:** Univariate analysis of clinicopathological factors for disease-specific survival

Variable	*n*	Hazard ratio	95 % CI	*P* value
Age (years)				0.854
<60	43	1		
≥60	43	1.074	0.504–2.284	
Gender				0.892
Male	63	1		
Female	23	0.942	0.398–2.23	
Tumor size (cm)				0.698
<4	31	1		
≥4	55	0.859	0.399–1.852	
Invasion depth				0.655
1,2	16	1		
3,4	70	0.813	0.328–2.015	
Distant metastasis				0.0005*
Absent	36	1		
Present	50	8.444	2.535–28.13	
Tumor stage				0.015*
I,II	23	1		
III,IV	63	5.996	1.418–25.34	
RP11-462C24.1				0.002*
Low	54	1		
High	32	0.044	0.006–0.322	

* *P* < 0.05

**Table 4:** Multivariate analysis of clinicopathological factors for disease-specific survival

Variable	Hazard ratio	95 % CI	*P* value
Distant metastasis (present/absent)	4.983	1.287–19.292	0.020*
Tumor stage (III,IV/I,II)	1.870	0.369–9.472	0.449
RP11-462C24.1 (high/low)	0.056	0.007–0.420	0.005*

* *P* < 0.05

## Discussion

Colorectal cancer is a common malignancy tumor, which on the top in the new cases in China [24]. Despite curative surgical resection of the primary tumor, 40–50 % of the patients ultimately die of metastasis [25]. However, the existence of multiple known carcinogens and varying genetic backgrounds makes it difficult to determine, which factors are most important in the development of CRC. Although many molecular markers, including carcinoembryonic antigen (CEA), have been exploited for CRC diagnosis, they lack sensitivity and specificity of evaluating the prognosis of CRC patient [31]. Thus, it is crucial to identify new biomarker responsible for predicting metastasis of CRC. In this study, firstly, we roughly screened aberrantly expressed lncRNAs between CRC tumor tissues and adjacent normal tissues and between metastatic CRC tumor tissues and non-metastatic CRC tissues by microarray assay. Six aberrantly expressed lncRNAs were then selected for their expression levels and genomic locations. The expression levels of those six lncRNAs were validated in another cohort of 86 patients with CRC by RT-PCR assay. We are particularly interested in one of those six lncRNAs, RP11-462C24.1, because its expression level was decreased with the malignant degree of CRC increased. Based on RT-PCR data of 86 patients, we investigated the association of RP11-462C24.1 expression level with clinicopathological characteristics and prognosis in CRC. To our knowledge, this is the first time to report the dysregulated expression pattern of RP11-462C24.1 in CRC. More important, we found that RP11-462C24.1 expression level was significantly associated with the state of patients’ distant metastasis (*P* = 0.028) and patients’ survival. Furthermore, patients with low RP11-462C24.1 expression level have poor prognosis.

The RP11-462C24.1, a type of lncRNA consisting of four exons with a length of 1,136 bp, locates in chr4q25. Ribosomal Protein L34 (RPL34), a leukemia-associated protein, is located head to head with RP11-462C24.1 [32]. It was reported that RPL34 was up-regulated in metastatic breast cancer [33]. RPL34 was also shown highly expressed in the colon cancer line RKO (a human colon cancer line). Thus, RPL34 was reported as a driver gene to promote RKO cell line proliferation [34]. However, RP11-462C24.1 was shown to be down-regulated in CRC in this study. Thus, it indicated that RP11-462C24.1, as an antisense transcript of RPL34, may involve in metastasis of CRC by negatively regulating RPL34 expression at the level of chromatin modification, transcription and posttranscriptional processing [10].

LncRNAs may function as oncogenes and tumor suppressors in cancer just like protein-coding genes. A tumor suppressor gene is broadly thought to normally inhibit tumor initiation and progression. And inactivation of tumor suppressor is prone to tumorigenesis. Several studies have demonstrated the potential role of lncRNAs as tumor suppressors. The maternally expressed gene 3 (MEG3) was the first lncRNA proposed to function as a tumor suppressor [35]. The MEG3 gene was detected in many normal human tissues, whereas it was not detected in any nonfunctioning adenomas of gonadotroph origin nor in a range of human cancer cell lines [36–39]. Moreover, ectopic expression of MEG3 was found to suppress the growth of several human cancer cell lines, further supporting the role of MEG3 as a tumor suppressor [36]. Functionally, MEGs has been implicated to interact with p53, a well-known tumor suppressor gene, and inhibit cell proliferation in vitro [36, 40]. Recently, LOC285194 was reported as a potential tumor suppressor in CRC. Its expression level was significantly lower in CRC and cancer cell line [41]. In present study, we found the expression level of RP11-462C24.1, a lncRNA whose function was previously unknown, decreased as the malignant degree of CRC increased, which indicated RP11-462C24.1 was a potential tumor suppressor lncRNA.

Though some LncRNAs have reported involving in the progression and development of tumors, the underlying molecular mechanism is still unclearly elucidated. Therefore, in the future, we will investigate the mechanisms of the low expression of RP11-462C24.1 in CRC, and whether and how it functions as a tumor suppressor gene.

In conclusion, we reported a novel dysregulated lncRNA in CRC, RP11-462C24.1, whose expression decreased with the development of CRC. The expression of RP11-462C24.1 has a good potential to distinguish CRC tissues from normal tissues, expressed by the large AUC of ROC (0.76). RP11-462C24.1 expression can also distinguish CRC tissues with metastasis from CRC tissues without metastasis, in which AUC of ROC is 0.62. Additionally, we revealed that RP11-462C24.1 expression significantly correlated with distant metastasis. We also demonstrated that RP11-462C24.1 expression was a predictor for patients’ survival as implied by Kaplan–Meier analysis. Moreover, univariate analysis revealed that three clinicopathological factors of patients with CRC: metastasis, tumor stage, and RP11-462C24.1 expression were significantly associated with patients’ survival. However, only metastasis and RP11-462C24.1 expression were significant in multivariate analysis, which indicated that RP11-462C24.1 expression was an independent prognostic indicator for survival of patients with CRC in addition to distant metastasis.

## Electronic supplementary material

Below is the link to the electronic supplementary material.

Supplementary Material 1: RP11-462C24.1 RNA sequence. (DOCX 13 kb)

Supplementary Material 2: Clinicopathologic characteristics of cohort of 86 patients. (XLSX 18 kb)

Supplementary Material 3: Primers used for Q-RT-PCR. (XLSX 11 kb)
