# Supplementary material for: Low expression of novel lncRNA RP11-462C24.1 suggests a biomarker of poor prognosis in colorectal cancer
Source: Med Oncol. 2014 Jun 8;31(7):31. doi: 10.1007/s12032-014-0031-7 (PMC4079943; doi:10.1007/s12032-014-0031-7)
Supplement: Supplementary file 1 — Supplementary Material 1: RP11-462C24.1 RNA sequence. (DOCX 13 kb) [file 12032_2014_31_MOESM1_ESM.docx]

RP11-462C24.1（1136bp）gene symbol :RPL34-AS1-002

AACCAACGTGCCGCCCCTCTGTGGGCGGGGATTCACACTAGCTGTCTGTGTCAAGGCCACGCAGAAATCTATTGGAAATCGCTGTGGAAACAAACGCAAATGAAAAGCCGCAAAGAGCAAAGGCTGCTCACTGGCCCGTCTCCTCTCATGACTGCTGCTCTGGGAACCACCTCTCTGCTCTCCATCGCTACCACTCTAGCCTGTGGCCTGCTCCTGGTTGAAGAAGCCATCAATGAAAATGAATACCACTCTGGAAATCTTGGGTGGTCTGACCTGATATTTTCGCATAGTGGCCACAGATATTTTAGGCCCCCAAGAGGTAGTCTTACTCTTTCAATTTTACTGGCTCCACTGTTTGAGGAACTTGGAACAATTTCCCCAGGGACTGGTCTGGAGAGACAGGATCTCACTATGTTGCCCAGGCTGGTCTCGAACTCTTGGACTCAAGTGATCCTCCTGCCTTGGCCTCCCAAAGTGCTGGAATTACAGGAATTAGCCTCCTTACTGTCAGACCAGCCACAGAACTTCCTCCCACTTATCCCTTAGATGTCAACTACCCACACTTCAAATCACCCTGGAGACTGATGTGACTGGGATGATTTGACTGCATGTAGGTCAGAGCTGCCTCCAGGCAACTTGTGGGGCCAAGGCTCTGTGGCTAGAAGGAAGTGAGCATTAATACATCACTTAGAACAGCAAAAATCATCATCTGAAGATCATCATCTGAAAAGATCAGCATCAGAAGAGCTGTGAATATCACCGATCTGATAATTGGGTATTTTTGGTAAAACACACACACACACACACACACACACACACACACACACACACGCACAGATGCACACAGCCCTGCTTTTATGAGAATTGAGCTTAAATAGATGTGGGTGGAGGTTTTACTGAACTGGATATTTCTTAACTTTTGGAGTACAACACCTAGTCTCCTAATTCCTGTCCTGCATAGCGTGCTTACACAGTATTTTAAAATTAGAGATAATTAAATTTGATTTTGTTTCAATAATTATTTTGACAGTTTTAAAATATCTTTTGGAATTGTTTGTACCATTCCAGTCCTTTTCAAATCCAAGACTGAAATGAATTTTTCTAGCTATTTATCCCATCTTTGAGTTAAGAACATA
